# Effects of an exercise programme for chronically ill and mobility-restricted elderly with structured support by the general practitioner's practice (HOMEfit) - study protocol of a randomised controlled trial

**DOI:** 10.1186/1745-6215-12-263

**Published:** 2011-12-21

**Authors:** Timo Hinrichs, Anna Moschny, Michael Brach, Stefan Wilm, Renate Klaaßen-Mielke, Matthias Trampisch, Petra Platen

**Affiliations:** 1 Department of Sports Medicine and Sports Nutrition, University of Bochum, 44780 Bochum, Germany; 2 Institute of Sport and Exercise Sciences, University of Muenster, 48149 Muenster, Germany; 3 Institute of General Practice and Family Medicine, University of Witten/Herdecke, 58448 Witten, Germany; 4 Department of Medical Informatics, Biometry and Epidemiology, University of Bochum, 44780 Bochum, Germany

**Keywords:** general practice, chronic disease, aged, mobility restriction, rehabilitation, physical activity

## Abstract

**Background:**

Exercise programmes can be administered successfully as therapeutic agents to patients with a number of chronic diseases and help to improve physical functioning in older adults. Usually, such programmes target either healthy and mobile community-dwelling seniors or elderly individuals living in nursing institutions or special residences. Chronically ill or mobility-restricted individuals, however, are difficult to reach when they live in their own homes.

A pilot study has shown good feasibility of a home-based exercise programme that is delivered to this target group through cooperation between general practitioners and exercise therapists. A logical next step involves evaluation of the effects of the programme.

**Methods/design:**

The study is designed as a randomised controlled trial. We plan to recruit 210 patients (≥ 70 years) in about 15 general practices.

The experimental intervention (duration 12 weeks)-a multidimensional home-based exercise programme-is delivered to the participant by an exercise therapist in counselling sessions at the general practitioner's practice and on the telephone. It is based on methods and strategies for facilitating behaviour change according to the Health Action Process Approach (HAPA). The control intervention-baseline physical activities-differs from the experimental intervention with regard to content of the counselling sessions as well as to content and frequency of the promoted activities.

Primary outcome is functional lower body strength measured by the "chair-rise" test. Secondary outcomes are: physical function (battery of motor tests), physical activity (step count), health-related quality of life (SF-8), fall-related self-efficacy (FES-I), and exercise self-efficacy (SSA-Scale).

The hypothesis that there will be differences between the two groups (experimental/control) with respect to post-interventional chair-rise time will be tested using an ANCOVA with chair-rise time at baseline, treatment group, and study centre effects as explanatory variables. Analysis of the data will be undertaken using the principle of intention-to-treat.

**Trial registration:**

Current Controlled Trials ISRCTN17727272.

## Background

### Health, physical functioning and physical activity in old age

Health status decreases with age. As an example, while about 40% of the German population aged 40 to 54 years suffer from at least two chronic diseases, this percentage rises to about 80% in the population aged 70 to 85 years. About 5% and 25% respectively suffer from five or more diseases in these two age groups [1]. Internationally reported frequencies of adults aged over 65 years with at least two chronic diseases vary between 40% and 80% [2-4]. Regular physical activity and exercise not only help to prevent the development of chronic diseases such as cardiovascular disease, osteoporosis, type 2 diabetes or obesity [5], but are also administered therapeutically [6,7].

Physical functioning is mainly determined by the following physiologic parameters: strength, endurance, balance and flexibility. All these parameters decline with age and show a clear relationship to everyday functioning and to the occurrence of falls [8-15]. Furthermore, a positive correlation between functional mobility (leg strength, balance, locomotion speed) and behavioural mobility (frequenting certain neighbourhoods and places inside and outside town) has been demonstrated [16]. As elderly people are able to compensate for the decline in their physiologic abilities for a long time (e.g. by using their arms when getting out of a chair), many elderly people function dangerously close to their maximum capacity [e.g. [17]]. Minor deterioration in their health status can lead to inability to perform everyday activities and consequently to a loss of independence [18]. Regular physical activity can delay the onset of functional decline [19]. Numerous interventional studies have shown effects of exercise on endurance, strength, balance and flexibility, even in mobility-restricted, frail or chronically ill older adults [e.g. [20]].

As a consequence of the existing evidence, present guidelines recommend multidimensional activity programmes, which should include daily training of endurance, strength, balance and flexibility, integrating preventive and therapeutic aspects [7]. Although the benefits of regular physical activity are widely known, most elderly individuals do not follow these recommendations [7,21-23]. Furthermore, compared to healthy persons, physical activity is even less prevalent among persons affected by chronic diseases or functional limitations [24-27].

### Defining the target group for an exercise intervention

To reach a certain population for an exercise intervention, the target group has to be analysed carefully. The target group of "older adults" is very heterogeneous with respect to individual abilities and needs. At the one end of the spectrum, there is a group of healthy, active and mobile seniors who organise their everyday lives independently, pursue their interests, and are integrated into social networks. At the other end, there is a group of chronically ill, demented, immobile and/or frail elderly individuals who have already lost their independence and live in special residences or nursing homes. The first group can be accessed by public activity offers or by group interventions in fitness facilities, community or geriatric centres (e.g. [28,29]). The latter group can be accessed by going into their residences and recruiting them for on-site exercise (group) interventions (e.g. [30]). On the other hand, there is also a large group of older people who are between those two extremes: sedentary, chronically diseased and/or mobility-restricted older adults who still live in their own homes. Those people are at high risk of losing their independence. They are difficult to reach for exercise interventions, as many of them rarely leave their homes [31]. Additionally, their own perception of limited health is a major barrier to engagement in physical activity [32-34]. Very few studies have tried to reach this group for exercise interventions.

### Reaching the target group

One possible way to reach sedentary or mobility-restricted community-dwelling elderly is to visit their homes. As an example, Tudor-Locke et al. [35] delivered an exercise intervention to older adults through existing home support infrastructures. The trained home support workers gave exercise instructions to their clients and provided encouragement during regular home visits. McMurdo and Johnstone [36] investigated a similar concept in which physiotherapists visited the residents of sheltered housing complexes at home and instructed them in home-based exercises.

Another way to approach the target group is to make use of the primary health care setting. This approach offers the following benefits:

(1) Access: apart from relatives or neighbours, one of the few persons who have regular access to the target group is the general practitioner (GP). A high percentage of the elderly population (e.g., 96% of 65- to 84-year-old participants in the German National Health Interview 2003 [37]) have a GP that they regularly consult for health problems.

(2) The trust level: GPs often have established long-lasting relationships with their older patients. The trusting relationship between physician and patient plays an essential role in patient compliance [38-40].

(3) Assessment of exercise eligibility: the GP is able to judge the patient's ability to perform an exercise programme. If necessary, additional examinations (e.g., exercise electrocardiograms, radiological examinations of joints or spine) can be performed or initiated to tailor the exercise programme to the patients' abilities and to avoid harm.

In summary, the GP's practice would appear to offer an ideal venue for recruiting and supporting patients to be physically active ("practice approach").

### Development and evaluation of the HOMEfit concept

With the considerations mentioned in the preceding sections in mind, the aim of our study group is to develop and evaluate a concept that delivers multidimensional exercise (consisting of strength, endurance, balance, and flexibility training) to chronically ill and mobility-restricted community-dwelling elderly by approaching and supporting them via their GP's practice. Based on a framework for evaluation of complex interventions developed by the Medical Research Council (MRC, United Kingdom) [41], a research plan was set up that contains the following four phases: (a) development, (b) feasibility, (c) evaluation, and (d) implementation.

The development phase included a literature review [42] and analyses of GP counselling behaviour in a cohort of older primary care patients [43]. The literature review revealed that even though there is conflicting evidence regarding the effectiveness of GP counselling on physical activity, the "practice approach" seems to be widely accepted. However, the GP should be supported by additional personnel and GP advice should be combined with behavioural interventions [42]. The epidemiological analyses revealed that only about one-third of all interviewees had been advised by their GP to get regular physical activity within the previous year [43]. This rate is consistent with international studies [44-48]. There may be many different reasons for rather low counselling rates across studies. Among others, the following barriers to GPs for counselling about physical activity have been reported: lack of educational resources and of formal clinician training for physical activity counselling, and time constraints during consultations [47,49-52]. Again, these findings support the idea of reducing the burden on GPs by including qualified personnel (e.g. an exercise therapist) in the counselling process.

Based on these literature results, a new kind of collaboration between GPs and exercise therapists has been set up, in which patients are recruited and assessed for eligibility by their GP [53]. The 12-week programme consists of multidimensional home-based exercise delivered to the participant by an exercise therapist in counselling sessions at the GP's practice and on the telephone. The exercise programme is imbedded in a behavioural intervention based on the Health Action Process Approach (HAPA) by Schwarzer et al. [54]. This model comprises aspects of well-established continuous and stage models that undertake to explain the process of behaviour change, and intends to overcome some of the limitations inherent in other models. The HAPA thus provided the basis for conceptualising and implementing a high-quality intervention fostering the initiation and maintenance of the multidimensional exercise programme.

In a pilot study, all predefined criteria for feasibility (adoption, safety and continuing participation) of the concept were met [55]. This permitted the decision to proceed in the research plan and to enter the next phase of research, which is "evaluation". The present study is the first study of the evaluation phase, which aims to assess effects of the new programme on physical function, physical activity, health-related quality of life, fall-related self-efficacy, and exercise self-efficacy.

### Main hypothesis

The experimental intervention (multidimensional home-based exercise with structured support given by the GP's practice) is more effective for increasing functional lower body strength (measured by a timed test of five sit-to-stand cycles ["chair-rise" test]) than the control intervention after 12 weeks.

## Methods/design

### Study overview: target group, setting, design and registration

**Figure 1:**
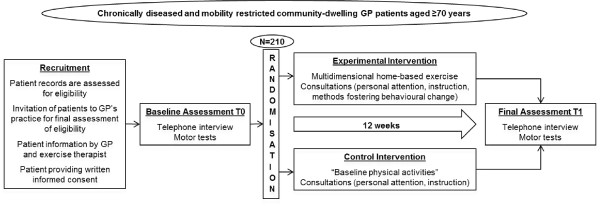
**Flow chart of the study, referring to the participant level**.

The study targets community-dwelling, chronically ill, and mobility-restricted patients, aged 70 years or above, who visit their GP's practice. The study aims to include 210 patients from about 15 GP practices. The settings of the intervention are (a) the practices, where consultations take place, and (b) the participants' homes, where the exercises are conducted. The study is part of the "evaluation" phase of the MRC framework [41]. It is a prospective randomised controlled trial and has been registered with the ISRCTN register managed by Current Controlled Trials http://www.controlled-trials.com. The registration number is ISRCTN17727272. For a flow chart, see Figure 1. Scientific lead, study management and overall coordination are performed by the Department of Sports Medicine and Sports Nutrition, University of Bochum.

### Recruitment

#### Recruitment of practices

About 15 GP practices are recruited from a regional network of research practices that is administered by the Institute of General Practice and Family Medicine, University of Witten/Herdecke. GP practices are chosen from urban and rural areas within a radius of 30 kilometres around the city of Bochum. Practices must be able to provide a separate room where assessments and intervention take place. In addition, they must be able to perform an electronic search within their patient files. Practices are included regardless of the number of GPs working within the practice. Practices that participated in the feasibility trial are excluded from participation.

#### Recruitment of exercise therapists

To be eligible for the study, exercise therapists must either hold a university degree (bachelor's or higher) in sports science (preferably with a specialisation in "prevention and rehabilitation") or in physiotherapy or have completed a three-year vocational training programme in physiotherapy (including the final state examination). All therapists have to complete an additional training programme for the HOMEfit study.

#### Recruitment of participants

210 participants (about 14 patients per GP practice) are recruited through GP practices. In short, a practice nurse (working at the respective practice) and a study physician assess the patient records for inclusion and exclusion criteria. Patients who seem to be eligible based on their clinical records are invited for a personal assessment (performed by the GP and an exercise therapist) of final eligibility at the GP's practice.

In the following steps of the recruitment procedure, the number of patients in each group is documented (for an overview see Table 1):

**Table 1:** Recruitment of participants

Step	Selection criteria	Documented variables	Performed by
**1**.	Patients ≥ 70 years who have seen their GP within the past 6 months → N1	Number (N1)	
	
**2**.	Screening N1: Exclusion of institutionalised patients (nursing home) → N2	Number (N2)	
	
**3**.	Screening N2 for inclusion and exclusion criteria → N3	Number (N3)	Study physician and practice nurse
			
	if N3 is < 20 → Exclusion of practice		
			
	if N3 is between 20 and x* → N3 = N4 → Step 4		
			
	if N3 is > x* → x* patients are randomly selected → x* = N4 → Step 4		
	
**4**.	Final list of patients to be invited → N4	-Number (N4) -Year of birth -Sex	

**5**.	Mailing of invitations to N4 for a screening at the GP's practice; appointments with interested patients are made according to a given timetable.		Practice nurse

**6**.	Screening of patients at the GP's practice → N5 -final assessment of eligibility -patient information -written informed consent	-Number (N5) -Year of birth -Sex -Written informed consent -Reasons for exclusion	1. GP 2. Exercise therapist

**7**.	Patients who keep their first appointment with the exercise therapist to start the intervention → N6	-Number (N6) -All patient characteristics	Exercise therapist

* X is set to 70 for the first practice and may be adapted during the course of the study depending on the recruitment success of the following steps.

*Step 1*. Number (N1) of patients ≥ 70 years who have seen their GP at least once within the previous six months. This number can be extracted easily from the usual electronic patient documentation systems. This search is performed by a study physician and a practice nurse.

*Step 2*. Number (N2) of community-dwelling patients out of N1. The study physician and the practice nurse screen N1 to exclude institutionalised patients (based on their residence address and the information provided by the practice nurse).

*Step 3*. Number (N3) of the patients out of N2 who have been judged as eligible (based on inclusion and exclusion criteria) by the study physician and the practice nurse after the assessment of the clinical records. If N3 is lower than 20, the respective practice is excluded from participation in order to conserve resources.

*Step 4*. Number (N4), year of birth and sex of the patients who receive an invitation letter. As the intervention only takes place once per practice, we aim to include between 8 and 20 (ideally 14) patients per practice out of N3. To prevent more than 20 patients from having to be included in the intervention at the respective practice, the number of patients who receive an invitation letter is limited to a maximum of x patients. x is set at 70 at the first practice. If N3 is higher than x, the x patients who receive an invitation are randomly selected from N3 by the study physician. The ratio of invited patients to patients who are ultimately included in the intervention is monitored. x may be adapted within the course of the study depending on the recruitment success of the following steps.

*Step 5*. In this step, invitation letters from the respective practice are mailed to the patients (N4) by the practice nurse. The nurse has to complete a prepared letter by inserting the patients' name and address, the GP's stamp and signature. Patients do not receive an additional phone call. A patient who is not able to make an appointment for the personal assessment within the predefined time periods (when the exercise therapist is present at the GP's practice) has to be excluded from participation. The assessments take place within the GP's usual consultation hours. However, practice personnel ensure that the prospective study participants do not have unnecessary waiting times.

*Step 6*. Number (N5), year of birth, sex, written informed consent (yes/no), and (where applicable) reasons for exclusion of the patients out of N4 who come to the personal assessment of final eligibility in the GP's practice. All of these patients get in contact with both the GP (1^st^) and the exercise therapist (2^nd^) the same day. The GP rechecks the medical exclusion criteria and informs the patient about essential features of the study. The GP confirms by signature that there are no medical reasons for excluding the patient. The exercise therapist checks for (and documents) the other inclusion and exclusion criteria, informs the patient about the intervention, and uses motivational interviewing techniques [56] to foster his/her intention to participate. Patients who meet all inclusion criteria, do not meet any exclusion criteria, and are willing to participate provide their written informed consent. Patients who have to be excluded and patients who refuse to participate are asked to give written consent for the use of these data in the recruitment analysis. Patients who have to be excluded receive alternative offers.

*Step 7*. Number (N6) and all patient characteristics (see "Baseline data" section) of the patients out of N5 who keep their first appointment with the exercise therapist to start the intervention.

#### Inclusion/exclusion criteria

This study aims at tertiary prevention and thus includes chronically diseased and mobility-restricted persons. The GP, who is familiar with the participant's medical history and his/her current health status, decides whether the patient is able to perform the exercise programme. Only individuals that are at high risk of adverse events are excluded for medical reasons.

##### Inclusion criteria

To be eligible for this study, patients have to be community dwelling (not institutionalised) and aged 70 years or above.

They have to be diagnosed with at least one of the following chronic diseases (according to the International Classification of Diseases): essential hypertension (I10.-), non-insulin-dependent diabetes mellitus (E11.-), chronic ischaemic heart disease (I25.-), heart failure (I50.-), atherosclerosis of arteries of extremities (I70.2-), chronic obstructive pulmonary disease (J44.-), chronic kidney disease (N18.-), spinal osteochondrosis (M42.-), coxarthrosis (M16.-), gonarthrosis (M17.-), osteoporosis with or without pathological fracture (M80.- or M81.-); **and **mobility limitation (according to the International Classification of Functioning, Disability and Health). Following the example of Rasinaho et al. [32], mobility limitation is defined using the following questions: "Are you able to walk two kilometres?" and "Are you able to climb one flight of stairs?" The response options are "Yes, with no difficulty"; "Yes, but with some difficulty"; "Yes, but with a lot of difficulty"; "Yes, but not without help"; and "Not even with help". Those who report at least some difficulty in either walking 2 km or climbing one flight of stairs fulfil the inclusion criterion. Those who report no difficulty in walking 2 km and climbing one flight of stairs are rated as having no mobility limitation and therefore are excluded from participation by the exercise therapist in step 6 of the recruitment process.

Participants have to at least be able to walk short distances indoors with or without a walking aid; but without the help of another person. Persons who are wheelchair bound are excluded.

Participants have to be able to visit the GP's practice for repeated consultations.

All participants need medical clearance from their GP (in step 6) to participate in the study. Furthermore, participants have to be able to cooperate appropriately and to follow the instructions of the home-based exercise programme (according to their GP's judgement). Finally, all participants have to provide written informed consent.

##### Exclusion criteria

Patients are excluded if they are unable to attend or complete the proposed course of intervention and follow-up. This includes not having a telephone and being unable to have telephone conversations.

Spouses of participants, persons living in the same household and former participants of the feasibility trial are excluded.

Participants who are unable to perform the chair-rise test (primary outcome) are excluded. This is tested by the exercise therapist within the recruitment process (step 6).

To be eligible for the study, participants have to be only moderately physically active or sedentary. Patients who report that they regularly perform exercises, sporting activities or leisure activities that cause sweating and/or harder breathing for 2 hours or more per week are excluded. Patients who report that they walk outdoors for 4 hours or more per week are also excluded.

Patients are excluded for medical reasons if they have untreated arterial hypertension or significantly elevated blood pressure despite antihypertensive medication (GP's judgement), higher-level chronic heart failure (New York Heart Association (NYHA) class III-IV), higher-level chronic obstructive pulmonary disease (Global Initiative for Obstructive Lung Disease (GOLD) stage IV), an acute psychiatric disorder (e.g. severe depression), or an advanced terminal illness. Furthermore, patients are excluded if they have suffered a clinically relevant cardiovascular event (e.g. unstable angina pectoris, myocardial infarction, coronary angiography and/or angioplasty), a clinically relevant cerebrovascular event (e.g. stroke, recurrent TIA), or deterioration of insufficiently controlled diabetes (according to their GP's judgement) within the previous 3 months, or if their HbA1c exceeds 10% (if available). Additional exclusion criteria are on-going rehabilitation measures following an inpatient surgical procedure and concurrent participation in another clinical trial.

#### Time schedule

The duration of the entire project is 36 months (December 2010 to November 2013).

The period between recruitment of a GP and patient screening at the GP's practice should ideally not exceed 8-10 weeks. For this reason, recruitment of GPs is performed in stages. Recruitment of GPs starts in August 2011 and ends in December 2012.

Recruitment of participants starts in January 2012 and ends in March 2013 (15 months). The last participant is expected to finish the intervention in June 2013. Altogether, the period between "first patient in" and "last patient out" is 18 months.

### Experimental and control intervention

#### Overview

The experimental intervention has been designed according to state-of-the-art physical activity recommendations for older adults [7]. It consists of:

1. multidimensional (strength, endurance, balance, flexibility) home-based exercise integrating preventive and therapeutic recommendations

2. consultations provided by an exercise therapist (at the GP's practice and via telephone) including personal attention, instruction, and methods fostering behavioural change.

The control intervention promotes baseline physical activities [[57], page 2], and thus consists of:

1. baseline activities of daily life, e.g. light-intensity walking,

2. consultations provided by an exercise therapist (at the GP's practice and via telephone) including personal attention and instruction.

**Figure 2:**
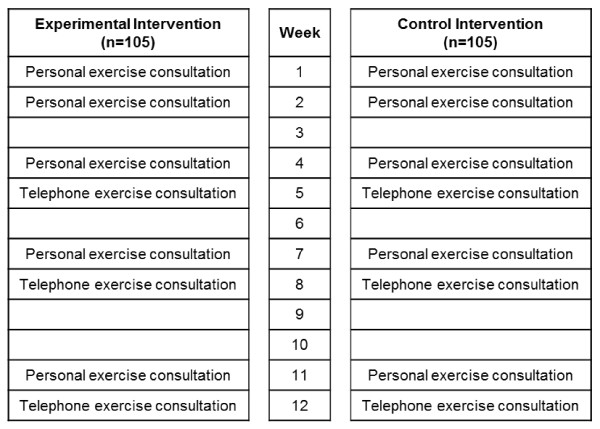
**Time schedule of the intervention**.

The duration of the intervention is 12 weeks (see Figure 2). The exercise therapist provides a given number of personal consultations at the GP's practice (weeks 1, 2, 4, 7, 11) and a given number of consultations on the telephone (weeks 5, 8 and 12). If the participant misses an appointment, a new appointment is scheduled within the following days or weeks (as soon as possible).

#### Common aspects for both groups

Any additional medical treatment is permitted before and during the trial, with the exception of on-going rehabilitation measures following an inpatient surgical procedure (see exclusion criteria).

The exercise therapist will inform the GP about the initial assignment and further development of his/her patients in order to enable the GP to encourage and support his/her patients during normal office visits.

In order to ensure that both groups receive a similar amount of personal attention, the control group receives the same number of consultations at the GP's practice and via telephone as the experimental group (see Figure 2).

#### Experimental intervention details

Development and content of the experimental intervention have already been extensively discussed and presented in the study protocol of the feasibility study [53]. Therefore, the following descriptions are limited to information on modifications of the programme (based on results of quantitative and qualitative analyses) and additional background that has not yet been presented in detail (e.g. aspects on behaviour change).

##### Home exercises

Analysis of correctness of exercise performance revealed that some exercises were less appropriate than others, or were only performed by a small percentage of participants [58]. These exercises have been replaced by more suitable ones. An overview of the chosen exercises is given in Table 2.

**Table 2:** Overview of home-exercises

Category	**No**.	Exercise (variants)
**Strength**	**1**	Knee extension (seated/standing)
	
	**2**	Leg abduction (standing)
	
	**3**	Calf-raise (standing)
	
	**4**	Biceps curl (seated/standing)
	
	**5**	Upper back (seated/standing)
	
	**6**	Seated crunches

**Flexibility**	**7**	Seated sit-and-reach
	
	**8**	Upper back and chest stretch (seated/standing)

**Balance**	**9**	One-leg-stand
	
	**10**	Tandem stand (tandem walk)

##### Consultation sessions

Table 3 presents the eight sessions of the multidimensional home-based exercise programme. Each session has a certain topic matched with the mode of consultation. Face-to-face consultations deal with the topics "Getting started", "Strength", "Endurance", "Balance", and "Flexibility". In these sessions, participants practise the accurate performance of exercises after considering why and how they should exercise. Telephone consultations cover "Barriers and resources to physical activity", "Physical activity and specific medical conditions", and "Living an active life".

**Table 3:** Overview of the 12-week experimental intervention: the HOMEfit exercise programme

**Session no**.	1	2	3	4	5	6	7	8
**Intervention week no**.	1	2	4	5	7	8	11	12

**Type of contact**	face-to-face	face-to-face	face-to-face	by telephone	face-to-face	by telephone	face-to-face	by telephone

**Main topic**	**Getting started**	**Strength**	**Endurance**	**PA and specific medical conditions**	**Balance**	**Barriers and resources to PA**	**Flexibility**	**Living an active life**

**Beginning**	Motives for participation Current PA behaviour and exercise biography	Identification of state of health and feeling Action control-Reflection of the previous week(s) Clarification of session topic						

**Theoretical part**	General health benefits of PA, recommendations for health-enhancing PA Goal setting for the end of the intervention The programme, organisational aspects Begin exercising-hints and safety tips	Significance of strength for health and everyday life How to improve strength	Significance of endurance for health and everyday life How to improve endurance	Benefits of PA for prevention and treatment of a specific medical condition, e.g. coronary heart disease, diabetes, or arthritis (according to patient's choice)	Significance of balance for health and everyday life (fear of falling) How to improve balance	Identification of individual barriers and resources to physical activity (significance of social support) Strategies for dealing with barriers	Significance of flexibility for health and everyday life How to improve flexibility	Lifestyle PA Staying physically active Falling back into inactive behaviour

**Practice**	Body perception: Active standing Warm-up programme	Warm-up programme Strength exercises	Warm-up programme Short walking session outside		Warm-up programme Balance exercises		Warm-up programme Flexibility exercises	

**Conclusion**	Goal setting and action planning for the following week	Goal setting and action planning for the following two weeks	Goal setting and action planning for the following week	Goal setting and action planning for the following two weeks	Goal setting and action planning for the following week	Goal setting, action planning and coping planning for the following three weeks	Goal setting, action planning and coping planning for the following week	Goal setting, action planning and coping planning for the near future

PA = physical activity

##### Changing physical activity behaviour

As initially stated, the experimental intervention is based on the Health Action Process Approach (Schwarzer et al.) in order to foster patients' behavioural change. In the following sections, the essential elements of HAPA are briefly introduced, directly followed by a paragraph (→) describing how the respective element is considered in the intervention.

As a continuous model, HAPA suggests a distinction between motivational processes that lead to a behavioural intention and volitional processes of goal pursuit that end with successful performance. As a stage model, the behaviour change process subdivides individuals into non-intenders, intenders, and actors. Non-intenders do not have the intention to change their behaviour; they need to be motivated (motivational phase). Intenders have formed an intention but have not yet started to act; they require skills to translate their intention into action (volitional phase). Actors have already started to implement their intention; they need assistance to maintain their initiated behaviour (volitional phase). For details, see Schwarzer et al. [54]. The stage version intends to deliver starting-points for interventions.

→ In the experimental intervention, we adopt the HAPA model as follows: patients who give their written informed consent to participate are considered as individuals with an intention to change their physical activity behaviour, even though this intention may be weak. Therefore, the experimental intervention aims at:

(a) strengthening participants' motivation and thus the intention to exercise, and

(b) assisting intenders and actors in improving their volitional execution skills to adopt the exercise programme and to maintain the new behaviour, respectively.

Non-intenders are not considered in this study.

##### Motivational elements

An intention is considered to be the basic precondition for a behavioural change and has been shown to be associated with physical activity behaviour [59,60]. According to Schwarzer et al. [54], perceived self-efficacy and outcome expectancy are the two major components influencing the formation of an intention. The significance of outcome expectations and in particular, perceived self-efficacy for physical activity behaviour has been proved in a number of studies in healthy or chronically diseased (older) adults [61-69].

###### Self-efficacy

A person's confidence in his/her ability to perform a particular behaviour under various circumstances is called self-efficacy. It is considered to be the basis for all human motivation and action [70]. Self-efficacy is thus a core element of many behaviour change models and a basic construct throughout all phases of the HAPA [54].

There are certain strategies for positively influencing an individuals' belief in his/her competence to increase physical activity and to exercise. Lee et al. [64] present sources of self-efficacy beliefs that have proved to be useful in exercise interventions for older adults:

a) Performance accomplishments: the experience of success strengthens an individual's self-efficacy, while negative experiences weaken the belief in one's own abilities. Performance accomplishments are suggested to have the strongest impact on perceived self-efficacy. Particularly for older adults, it has been proposed to set achievable subgoals allowing for gradual progress.

b) Vicarious experience: "People with a comparable lifestyle [...] or those with similar characteristics [...] may serve as models for a specific behaviour and necessary skills. This may be a particular issue for older people where a lack of role models within a similar age group may reinforce the belief that exercise is irrelevant" [[64], page 1694]. For successful vicarious learning, a role model chosen for an older adult must be comparable with the subject. Seeing this role model exercise may strengthen the older person's belief in his/her own capabilities to perform equal activities.

c) Verbal encouragement: giving realistic positive feedback is a further strategy for increasing perceived self-efficacy. Encouragement from health care professionals has been shown to be particularly motivating among older adults. Interventions that used both telephone and face-to-face encouragement to support older people in regular walking as exercise significantly increased participants' exercise self-efficacy and self-reported amount of walking [71,72].

d) Perceived physiological and affective responses: exercising is accompanied by specific physiological and emotional responses that may be positive (e.g. vitalisation-enjoyment) or negative (e.g. fatigue, breathlessness, pain-stress, fear). Exercise interventions for older adults should enable the experience of positive physical and mental states and avoid physical overexertion and mental stress in order to promote self-efficacy beliefs and in so doing, foster the motivation to exercise. Older adults in particular may perceive non-hazardous physical symptoms (e.g. muscular fatigue) as signs of vulnerability. These older adults have to be assisted in reinterpreting what they perceive as health-threatening symptoms.

###### Outcome expectancy

Outcome expectancy is defined as the expectation of positive and negative outcomes that follow the accomplishment of a certain action [73]. Regarding exercise, such expectations may refer to physiological and affective responses, reactions from the social environment or (dis)satisfaction with the performance. Outcome expectations may be based on positive and negative experiences, a lack of experience, or attitudes towards exercise and physical activity. Firstly, interventions for older adults should aim at increasing the awareness of the multifaceted positive health outcomes of exercising in old age. Secondly, it is necessary to enable positive and avoid negative experience of physical activity (see strategy d) above). For details, see Williams et al. [73].

→ Implementation in the intervention: the overriding goal of each session of the experimental intervention is to positively impact perceived self-efficacy and outcome expectancy concerning physical activity and exercise. The individuals' motives for participation are discussed at the beginning of the first session. With regard to outcome expectancy, exercise therapists discuss the benefits of physical activity for health in general and for a specific medical condition (according to the patient's choice from a given set of medical conditions; session nos. 1, 4), and highlight the significance of physical competence (strength, endurance, balance, flexibility) for autonomous mastery of everyday life (session nos. 2, 3, 5, 7; see Table 3). The following sources are provided to increase participants' exercise self-efficacy: (1) exercise therapists tailor the programme to the individual capacities and needs of each participant (not only on the physical but also on the cognitive level) to avoid excessive demands and facilitate the experience of success; (2) throughout all sessions, exercise therapists listen with empathy and encourage participants; (3) a role model (older adult in street clothes performing the exercises of the programme) is shown in the illustrated workbook (see below: *Materials*). All these elements aim at increasing the motivation to perform the home-based exercise programme.

##### Volitional elements

In addition to strengthening the motivation to exercise, participants are assisted with strategies that facilitate the translation of their intention into action. Goal setting, strategic planning (action planning and coping planning) and action control have been shown to be effective self-regulatory skills that are particularly important in the first weeks of trying to overcome a sedentary behaviour. This also applies to older adults [54,59,60,74-79].

###### Goal setting

Setting a goal leads to a "sense of commitment that obligates the individual to realize the goal" [[80], page 494]. Proximal and specific goals result in better implementation than distal and vague goals. For details see the review of Gollwitzer [80].

→ Implementation in the intervention: at the beginning of the intervention, the exercise therapist clarifies the distal goal for the end of the intervention, which ideally corresponds to the physical activity recommendations. To increase and maintain participants' exercise self-efficacy, the distal goal is broken down into achievable subgoals. At the end of each session, participants are assisted in individually setting proximal goals for the next (two/three) week(s) of the intervention, e.g. performance of specific exercises on one day, walking for 15 minutes as exercise on two days of the following week (see Table 3).

###### Action planning

In "action plans", people define where, when and how they will perform a certain action. This leads to a more or less "automatic" initiation of the intended behaviour when the predefined situation occurs [54]. Action plans foster goal attainment by helping people get started [80].

→ Implementation in the intervention: action planning is a basic element at the end of each session of the intervention (Table 3). Participants plan on which days and at what time they will either go walking as exercise or do their strength, balance and flexibility exercises.

###### Coping planning

After people specify when, where, and how they intend to attain their goals, they need to anticipate possible barriers and to develop coping strategies [54]. Combined action and coping planning has been shown to be even more effective in increasing physical activity than action planning alone [76,78].

→ Implementation in the intervention: coping planning is addressed as a central topic of session no. 6 (see Table 3). During the telephone consultation, participants identify the individual barriers that prevent them from performing the planned exercises. Subsequently, they generate strategies to overcome these barriers. Coping plans are added on top of action plans at the end of session nos. 6 to 8.

###### Action control

"Self-monitoring is essential in action control as long as a behaviour has not become a habit. To control their behaviour individuals must monitor their actions to evaluate whether they are on track [...]" [[60], page 89].

→ Implementation in the intervention: patients are asked to fill out an activity log every evening. They record duration of walking as exercise, performance of home exercises, total step count (each participant gets a pedometer that displays daily step count on a screen), and comments (e.g. reasons for inactivity, problems with certain exercises). This self-monitoring facilitates matching of action plans and actual performance of the exercise programme. Additionally, it provides the basis for possible adaptations of exercises to individual requirements in the course of the programme. The activity log is discussed at the beginning of consultations during the reflection on the previous week(s) (see Table 3).

##### Resources and barriers

The implementation of volitional skills and goal pursuit are influenced by a variety of factors. There may be barriers and impediments to physical activity, but also support and resources [54]. Beside self-regulatory skills and (exercise) self-efficacy, social support has a high impact on physical activity behaviour. Significant others, such as health care professionals, exercise instructors, family members or friends, may positively influence the initiation and maintenance of a physically active lifestyle [81].

→ Implementation in the intervention: the HOMEfit concept is based upon the structured support given by the GP's practice. The GP recommends and repeatedly encourages his/her patient to take part in the exercise programme. The exercise therapist provides individual consultations to foster patients' motivation and volitional skills to perform the home-based exercise programme.

Beyond this social support, session no. 6 is targeted towards discussing potential support from family members or friends and other resources for overcoming identified barriers to physical activity (see "Coping planning" and Table 3).

##### Materials (experimental intervention)

Each participant gets an illustrated workbook that contains information on each topic of the eight intervention sessions as well as instructions and pictures for all home-based exercises. Additionally, it provides worksheets for goal setting, strategic planning, and action control (activity log).

Participants are equipped with pedometers (Walking style Pro, Omron Healthcare Co., Kyoto, Japan). Furthermore, they receive elastic resistance bands for performing several home exercises (Thera-Band^® ^GmbH, Dornburg-Frickhofen, Germany). Two different levels of resistance, which are rather low within the available range, have been chosen for the HOMEfit programme: yellow ("thin") and red ("medium"). Every participant is provided with one yellow and one red band.

#### Control intervention details

The control intervention focuses on promoting baseline activity. Baseline activities are defined as "light-intensity activities of daily life, such as standing, walking, and lifting lightweight objects" [[57], page 2]. An increase in these activities is not expected to result in the same health benefits brought about by an exercise programme or walking as exercise. However, minor positive effects (due to a reduction of sedentary time and an increase in total energy expenditure) still seem possible, especially in previously sedentary older adults.

##### Consultation sessions

Table 4 presents the eight sessions of the control intervention. The topics discussed in the initial face-to-face consultation are similar to the first session of the experimental intervention. During the second face-to-face session, exercise therapists discuss participants' daily routines with regard to their physical activity and sedentary behaviour. During the final personal consultation, participants once again reflect about their daily routine and current physical activity level compared to the level at the beginning of the study. The two blocks of personal and telephone consultations in between are dedicated to the discussion of opportunities for increasing baseline activity at home and in public, and implementation, respectively. Finally, participants are counselled by phone with regard to staying active in daily life.

**Table 4:** Overview over the 12-week control intervention

**Session no**.	1	2	3	4	5	6	7	8
**Intervention week no**.	1	2	4	5	7	8	11	12

**Type of contact**	face-to-face	face-to-face	face-to-face	by telephone	face-to-face	by telephone	face-to-face	by telephone

**Beginning**	Motives for participation	Identification of state of health and feeling						

**Topics**	Current PA behaviour and exercise biography General health benefits of PA Baseline PA The programme, organisational aspects	My daily routine and physical (in)activity	Opportunities for increasing baseline activity at home	Implementation of baseline activities at home	Opportunities for increasing baseline activity in public	Implementation of baseline activities in public	My daily routine and physical (in)activity: before and today	Staying physically active Falling back into inactive behaviour

PA = physical activity

There is no goal setting or strategic planning in the control intervention. Exercise therapists focus on giving information and instructions.

##### Materials (control intervention)

Each participant receives a booklet containing information on the benefits of physical activity for health and everyday life, opportunities to integrate more baseline physical activity into the daily routine, and on how to keep up. Participants receive no further materials.

### Study data

#### Baseline data

##### Characterisation of participating practices

Profile data of practices are evaluated by a self-report questionnaire completed during the initiation visit to the respective practice (urban or rural area; quarterly number of patients seen; number, age and sex of all involved practitioners). Questionnaires (paper protocols) are forwarded to the data manager.

##### Characterisation of exercise therapists

Date of birth, sex and vocational background (university degree/vocational training) of exercise therapists are evaluated by self-report questionnaire. Questionnaires (paper protocols) are forwarded to the data manager.

##### Characterisation of participants

Baseline data of participants are assessed within the baseline assessment (T0, see Figure 1). Socio-demographic data (including year of birth, sex, socio-economic status, living situation, social support) are assessed by computer-assisted telephone interview following German epidemiologic standards published by the Robert Koch Institute [82]. Physical activity is assessed by the use of the PRISCUS-Physical Activity Questionnaire (PRISCUS-PAQ), administered by telephone interview [83-85]. Current walking ability (no walking aid/cane/rollator) and frequency of falls (12-month recall) are also assessed by telephone interview. Falls are defined as "an unexpected event in which the participants come to rest on the ground, floor, or lower level" [86].

Weight and height are measured by standard protocol at the GP's practice by a trained assessor and electronically documented.

Chronic diseases (according to the inclusion criteria: essential hypertension, non-insulin-dependent diabetes mellitus, chronic ischaemic heart disease, heart failure, atherosclerosis of arteries of extremities, chronic obstructive pulmonary disease, chronic kidney disease, spinal osteochondrosis, coxarthrosis, gonarthrosis, osteoporosis with or without pathological fracture) are documented by the GP on a standardised paper protocol. Finalised protocols are forwarded to the data manager by fax. Originals remain in the practice (for monitoring purposes).

#### Efficacy data

Efficacy data are assessed within the baseline assessment (T0, before randomisation) and within the final assessment (T1, after 12 weeks of intervention, see Figure 1). Data from baseline and final assessment obtained by computer-assisted telephone interview are electronically documented. Data obtained within the motor tests at the GP's practice are documented on a standardised paper protocol. Electronic files and paper protocols are forwarded to the data manager.

##### Primary efficacy endpoint

The "chair-rise" test, a timed test of five maximum speed repetitions of rising from a chair (arms folded across the chest) and sitting down, is considered to be a measure of functional lower body strength and dynamic balance capability and serves as primary outcome (continuous variable) [87-90]. A test-retest (2 weeks) correlation of 0.73 has been reported for this measure [91]. The terms "chair stand" [91] or "sit to stand" [92,93] have also been used for this test.

Rising from a chair without hand use is a complex motor task that is challenging for many elderly individuals, in particular for those who suffer from neuromuscular or musculoskeletal impairments [94-97]. Lower body strength and reduced balance limit mobility (e.g., walking speed, independent ambulation, stair climbing) [92,98-100] and constitute important risk factors for falls [12] in older adults. Prospective cohort studies suggest that measures of lower extremity function may predict the future onset of disability [87,90], hospital [88] and nursing home use [101], and mortality [102].

The test is performed by a specially trained assessor at the GP's practice and is documented on a standard paper protocol. For methodological reasons, patients who are unable to perform the test have to be excluded from participation. Guralnik et al. [90] reported that in a sample of more than 5,000 community-dwelling older adults (71 years and older), 21.6% were unable to complete the test. However, within our feasibility study, only 3 out of 91 participants (3.3%) were unable to perform the test.

##### Secondary efficacy endpoints

Further physical functioning (mobility, aerobic capacity, strength, balance, and flexibility) is assessed by the use of different performance tests. All of these tests are performed by a trained assessor at the GP's practice in a given order. The "timed up-and-go" (TUG) is used to quantify mobility and coordination [103]. The time that the patient needs to rise from a chair, walk three metres, turn, walk back, and sit down again (one attempt) is taken. The test has been shown to be a sensitive and specific measure for identifying elderly individuals who are prone to falls [104]. The "2-minute step-in-place" test is used as a measure of aerobic endurance [105]. This test assesses the number of times, within two minutes, that the participant is able to step in place, raising his/her knees to a height halfway between the iliac crest and the middle of the patella (one attempt). Grip strength of the patient's dominant hand is measured using a Jamar hand dynamometer (Sammons Preston Inc., Bolingbrook, IL, USA) (best of three attempts) [106]. "Tandem stand" is used to assess standing balance. The patient's attempt to maintain his/her feet in the tandem position (heel of one foot directly in front of the other foot) is timed (maximum ten seconds, best of three attempts) [107]. Walking balance is assessed by "tandem walk". Patients are asked to attempt to walk with the heel of one foot directly in front of the other foot (maximum eight steps, best of three attempts). The "chair sit-and-reach" test (one attempt per leg) is used as a measure of hamstring flexibility [108].

Physical activity is estimated by the use of a pedometer (Walking style Pro, Omron Healthcare Co., Kyoto, Japan) and expressed as "mean steps per day". Participants are asked to wear the pedometer for a period of six days.

Health-related quality of life, fall-related self-efficacy ("fear of falling") and exercise self-efficacy are assessed by the use of questionnaires. The Short Form-8 Health Survey (SF-8™ 4-week recall version; computer-assisted telephone interview) that yields an eight-part profile of functional health and well-being is used to assess health-related quality of life. Two SF-8 composite scores are calculated: the SF-8 physical component score (PCS) and the SF-8 mental component score (MCS). The instrument has demonstrated good reliability and validity [109]. Fall-related self-efficacy ("fear of falling") is assessed by the Falls Efficacy Scale-International Version (FES-I; computer-assisted telephone interview) that has demonstrated high internal reliability and high test-retest reliability [110]. The confidence that one is capable of adhering to an exercise programme even under adverse conditions (exercise self-efficacy) is assessed by the "SSA-Scale" (according to Fuchs & Schwarzer [111]; self-administered questionnaire on paper protocols).

All secondary outcome measures result in continuous variables.

#### Patient compliance

Attendance at consultations (at the GP's practice and on the telephone) is documented by the exercise therapists as measure of patient compliance (paper protocols).

#### Appraisal by participants

During the final telephone interview, participants who completed the intervention are asked to rate the programme (standardised questionnaire). The rating (on a scale) includes quality, content and frequency of programme actions (e.g. personal and telephone exercise consultations) as well as quality and content of materials (e.g. workbook). Additionally, participants are asked to rate the concept used to approach and support them through their GP's practice.

#### Appraisal by GPs

After completion of the intervention within their practice, GPs are asked to rate the programme (standardised questionnaire on paper protocol). The rating (on a scale) mainly refers to the GPs' perception about their effort expended with and their benefit derived from the programme.

#### Appraisal by exercise therapists

After completion of all interventions, exercise therapists are invited to take part in a focus group discussion to share their opinion and experiences concerning the additional training programme for the HOMEfit study as well as the design, the content and the practical application of the HOMEfit programme.

#### (Serious) adverse events

During every encounter with the participant (either experimental or control intervention group), the exercise therapist assesses and documents all adverse events (AEs, any previously unknown or increasing symptom or disease of any participant) by a standardised report protocol. The therapist forwards all protocols with AEs (the same day, personally or via fax) to the GP, who judges: serious/not serious; related/not related to participation in the study; subject may not continue participation/subject may continue participation but has to pause completely for x days/subject may continue participation immediately but with restrictions for x days/subject may continue participation immediately without any restrictions. Adapting the ICH Guidelines on "Clinical Safety Data Management-Definitions and Standards for Expedited Reporting E2A" [112] to our intervention, a serious adverse event (SAE) is defined as "any untoward medical occurrence that [...] results in death, is life-threatening, requires inpatient hospitalisation [...]" or "results in persistent or significant disability/incapacity [...]".

Outcome assessors report SAEs to the GP on the same day, if they obtain the information during assessments at the practice.

SAEs are also independently reported by the GP using the standardised report protocol immediately after he/she obtains knowledge of them during routine consultations at the practice or via other information routes.

Protocols of adverse events that have been classified as "serious" by the GP are forwarded promptly by fax (secure receipt) to the SAE manager (who is affiliated to the Institute of General Practice and Family Medicine, University of Witten/Herdecke), who reviews the GP's judgements. AE protocols remain at the practice for monitoring purpose.

The Institute of General Practice and Family Medicine (University of Witten/Herdecke) collects all SAE protocols, analyses frequency and type of SAEs and provides summary reports to the Data Safety and Monitoring Board (DSMB) on a regular basis. In case of numerous occurrences of SAEs, the DSMB suggests that the study should be stopped.

#### Reasons for discontinued intervention

For future report in the CONSORT flow diagram [113], medical as well as non-medical reasons for discontinued participation (if applicable) are documented by the exercise therapist (paper protocol). For every participant, this documentation starts with the date on which written informed consent is given. Participants who temporarily or ultimately discontinue the participation voluntarily are asked to give a personal reason (voluntary response) by their therapist, either during a personal encounter or by telephone.

### Quality assurance and monitoring

All interventions, as well as the most important organisational issues, are documented in manuals and further documents that are used to advise and to train study personnel in a standardised way, especially those involved in data assessment or application of the study interventions. Results of quality checks are reported at regular staff meetings to allow feedback and refinements.

The state of practice recruitment, patient participation and drop-outs will be reported regularly to the project manager.

All monitoring procedures are laid down in a monitoring manual and administered in a standardised fashion. Monitoring physicians act independently of patients, GPs, exercise therapists, outcome assessors, telephone interviewers, data managers, and the project manager.

Monitoring visits to every participating practice are performed as an initiation visit at the recruitment of the practice, an unannounced visit in the period between first patient in/last patient out for the respective practice, and a final visit after last patient out. All visits to the respective practice will be performed by the same monitoring physician.

GP practice teams keep all information about each patient in an individual file. During monitoring visits, the files are checked for written informed consent of the patient, paper protocol of patient's chronic diseases, signed medical clearance by the GP, inclusion and exclusion details, dates of all face-to-face contacts of the exercise therapist and of the outcome assessor with the patient at the practice, and (S)AE reports. Any deviation from expected standards are reported to and discussed with the project manager.

Data completeness and plausibility monitoring and control of correct randomisation/allocation of patients will be performed by the data management at the Department of Medical Informatics, Biometry and Epidemiology, University of Bochum. Monitoring physicians and data managers will regularly exchange and document information about missing data and deviation from procedures.

The monitoring physicians will provide summary reports to the Data and Safety Monitoring Board (DSMB) about monitoring results and SAEs (see above) on a regular basis.

### Biometry

#### Data management

Data management is performed by the Department of Medical Informatics, Biometry and Epidemiology, University of Bochum.

#### Methods to prevent bias

The Department of Medical Informatics, Biometry and Epidemiology generates and holds the randomisation schedule. Randomisation is stratified by centre. Allocation concealment is ensured, as the randomisation code is not released until the patient's appointment with the exercise therapist to start the intervention (after informed consent has been obtained and all baseline measurements have been completed). The therapist receives the allocation information by fax.

Outcome assessors (including telephone interviewers), monitors, (S)AE manager and practice nurses are blinded with respect to group allocation. Exercise therapists (who deliver the programme), GPs (who motivate the patients in the course of the programme), and the project manager (whose responsibilities include the support of exercise therapists) are informed about group allocation.

After being allocated to one of the two interventions, participants get detailed information about their "own" intervention. Information about the content of the "other" intervention remains superficial to avoid bias by patient preference [114].

#### Sample size and power calculations

A feasibility study (single-arm interventional study [53]) has been conducted to allow for sample size/power calculation.

For sample size/power calculation- an ANCOVA (with chair-rise time at baseline, treatment group and study centre effects as explanatory variables and post-intervention chair-rise time as dependent variable) was used. It included the following steps and assumptions (unless otherwise noted, all numerical values were taken from the feasibility study):

1. Baseline chair-rise times were assumed to be normally distributed with mean 15.74 s and variance 25.09 s independent of group allocation.

2. The centre effect was modelled as a fixed normal random variable with mean 0 s and variance 1.59 s and added to the chair-rise time at baseline.

3. Post-intervention chair-rise times were assumed to be normally distributed with variance 25.09 s (again independent of group allocation) and correlated with baseline values (Pearson correlation of 0.78).

4. The difference in chair-rise time between the intervention and the control group at study end was assumed to be 2.0 s (improvement of 2.5 s in the intervention group and 0.5 s in the control group). This is a conservative assumption, as the feasibility study revealed an improvement of 2.93 ± 3.92 s for the intervention.

5. A drop-out-rate of 20% was assumed. This is a conservative assumption, because the feasibility study indicated an unadjusted drop-out-rate of 17% among participants who were able to perform the chair-rise test.

6. Missing values in both groups were each replaced by randomly selecting a change in chair-rise time from the control group and adding this difference to the baseline chair-rise time. This is a conservative approach, as it weakens the treatment effect of the intervention while simultaneously maintaining the control group effect.

Using these steps (1.-6.) the simulation (10,000 runs) with α = 0.05 revealed that 105 patients per group assure a power of 94.6%.

Making the even more conservative assumption that the correlation between chair-rise time at baseline and chair-rise time at study end is 0.7 instead of 0.78, the number of 105 patients per group still results in a power of 86.7%.

#### Statistical analyses

##### Baseline data

All baseline data are analysed descriptively and presented per group (experimental vs. control).

##### Primary efficacy endpoint

The hypothesis of differences between the two groups (experimental vs. control) concerning the post-interventional chair-rise time, is tested using an ANCOVA with chair-rise time at baseline, treatment group and study centre effects as explanatory variables. Analysis of the data is undertaken using the principle of "intention-to-treat" (ITT) which will be used as the official result of the study. Additionally, a secondary "per protocol" analysis is performed. Participants who abort the intervention are not invited for the final assessment. This will result in missing values. Missing values in both groups are each replaced by randomly selecting a change in chair-rise time from the control group and adding this difference to the baseline chair-rise time (see sample size calculation).

##### Secondary efficacy endpoints

Effects on secondary endpoints are analysed in the same manner as effects on the primary endpoint. Cases with a missing value at baseline are excluded for the respective analysis.

##### Patient compliance, appraisal by participants and by GPs, (S)AEs, discontinued participation

Patient compliance, appraisal by participants and by GPs, (S)AEs, and discontinued participation are analysed descriptively and presented per group (experimental vs. control).

### Ethical considerations and ethical approval

Compared to a sedentary lifestyle, exercise (especially walking outdoors) may initially increase the risk of musculoskeletal injuries. However, in the long term, regular exercise is considered to reduce fall and injury risk. The expected cardiovascular benefits of regular exercise exceed the risk of adverse cardiovascular events. To minimise the risk of adverse events, the exercise programme is tailored to the patient's abilities by a qualified exercise therapist in cooperation with the GP. Accident insurance is taken out for all participants. In case of numerous occurrences of SAEs, the DSMB will suggest that the study should be stopped.

All participants (patients, GPs), can discontinue their participation at any time without giving reasons. Patients are assured that they will be treated by their GPs in the same manner as before even if they discontinue participation.

The research is carried out in compliance with the Helsinki Declaration, the German Good Clinical Practice [115] and Good Epidemiological Practice [116] protocols. All data protection regulations of the State of North Rhine-Westphalia are followed. The protocol was approved by the Witten/Herdecke University Ethics Committee on 15 August 2011 (Reg.-No. 77/2011).

## Discussion

A physical activity programme for community-dwelling, chronically ill, and mobility-restricted patients has been developed, which: (a) on the individual level, contains multidimensional exercise (according to state-of-the-art guidelines) and consultations (including strategies fostering behavioural change), and (b) on the institutional level, establishes a cooperation between GPs and exercise therapists. After successful completion of a "development" and a "feasibility" phase, the present protocol describes the first study of the "evaluation" phase [41].

For this study, a measure of physical performance (namely functional lower body strength) has been chosen as primary endpoint. Using a physical performance test as the primary efficacy endpoint of an RCT makes it worthwhile to contemplate the position of physical functioning in different theoretical ability/disability frameworks. A traditional model developed by Nagi in 1964 [117] suggests that all disability originates from pathology or disease: disease leads to impairment, impairment to functional limitation and functional limitation to disability. Considering the evidence that physical inactivity or muscle disuse can be just as responsible for physical decline leading to disability [e.g., [6,118]], Rikli & Jones [105,119] recommended revising the traditional Nagi model by including "lifestyle/inactivity" as another possible origin of a disabling process. This revised model not only supports the role of physical inactivity in the loss of function (independent of the disease process), but also strengthens the role of physical activity/lifestyle interventions in the prevention of disability. WHO's International Classification of Functioning, Disability and Health (ICF) (which is the successor to the International Classification of Impairment, Disability and Handicap [ICIDH, [120]]) provides another, much more complex conceptual framework of functioning and disability [121]. The ICF defines "functioning" as a multidimensional (bio-psycho-social) concept relating to body structures and functions, activities, and participation. In addition, a new factor in comparison to former ability/disability models is that a person's functioning is always viewed in relation to health conditions and contextual factors (environmental and personal). "Disability", which is complementary to "functioning", encompasses any or all of the following: an impairment of body structure or function, a limitation in activities, or a restriction in participation. In contrast to earlier ability/disability frameworks, disability is not considered as an ultimate endpoint of a (mainly unidirectional) process. Rather than categorising people with disabilities as a separate group, disability is conceived as a continuum [122].

Referring to the ICF model and to a "conceptual description of the rehabilitation strategy" by Stucki et al. [123], which is based on this model, the primary target (and therefore a key outcome) of our intervention is optimal (physical) functioning. To achieve this target, "approaches to optimise a person's capacity" are applied and integrated: "approaches which build on and strengthen the resources of the person, which provide a facilitating environment, and which develop performance in the interaction with the environment" [[123], page 280]. The complexity of our intervention means that it can be expected to have diverse effects. This necessitates the use of several secondary outcome measures [114], not only on the physical, but also on the mental and behavioural level. Furthermore, quantitative and/or qualitative data concerning the appraisal of the programme will be obtained from all stakeholders.

The ultimate goal of our research is to guide policy-makers in planning health care services. In turn, one of their high priority goals is to optimally allocate limited resources. This will necessitate an economic evaluation of our programme, including cost-effectiveness and cost-benefit analyses [124-126]. Therefore, future RCTs will have to show the efficacy on endpoints such as hospital admissions, nursing home use, or utilisation of health care services. For the most cost-effective results, recruitment for the programme should then be focused on patients who will most likely benefit from it [127,128]. Consequently, GPs will have to be outfitted with simple screening tools to identify those patients and to be motivated and trained to use them.

In conclusion, the object of research is a home-based exercise programme that approaches and supports community-dwelling but mobility-restricted older adults via their GP and an exercise therapist with regard to effects on physical function, physical activity, health-related quality of life, fall-related self-efficacy, and exercise self-efficacy. If the project is successful, long-term effects on further endpoints (e.g. hospitalisation rate) and cost-effectiveness of the programme should be evaluated. The programme's high level of flexibility could facilitate future implementation as part of primary health care.

## Trial status

At the time of manuscript submission (15 November 2011), recruitment of GPs has started. Recruitment of participants has not started yet (see time schedule).

## Competing interests

The authors declare that they have no competing interests.

## Authors' contributions

TH and MB specified the research plan for development and evaluation of the HOMEfit concept. TH and AM initiated, planned and conceptualised the present study and applied for the research grant. All co-authors supported the grant application and substantially contributed to conception and design of the study by giving relevant intellectual input on all aspects of the study. TH drafted the manuscript; AM ("Experimental and control intervention", tables and figures), SW ("Quality assurance and monitoring"), and MT ("Sample size and power calculations") participated in drafting the manuscript. All authors revised the manuscript critically for important intellectual content. All authors approved the version to be published.
